# Functional Outcomes of Proximal Humeral Fracture Fixation Using a Proximal Humerus Internal Locking Osteosynthesis (PHILOS) Plate with Fibular Strut Graft Augmentation: A Retrospective Review

**DOI:** 10.7759/cureus.84172

**Published:** 2025-05-15

**Authors:** Nilesh Verma, Abhishek Sengupta, Hitesh Garg, Chandra Kant Singh, Siddhant Jain, Anurag Sharma, Zeeshan A Khalil

**Affiliations:** 1 Central Institute of Orthopedics, Vardhman Mahavir Medical College (VMMC) and Safdarjung Hospital, New Delhi, IND; 2 Orthopedics and Traumatology, Safdarjung Hospital, New Delhi, IND; 3 Orthopedics, Vardhman Mahavir Medical College (VMMC) and Safdarjung Hospital, New Delhi, IND; 4 Orthopedics, Safdarjung Hospital, New Delhi, IND; 5 Orthopedics and Traumatology, Vardhman Mahavir Medical College (VMMC) and Safdarjung Hospital, New Delhi, IND

**Keywords:** fibular autograft, osteoporotic fractures, philos plating, proximal humeral fracture, trauma

## Abstract

Introduction

Proximal humerus fractures (PHFs) are one of the most common fractures encountered in the emergency commonly affecting the elderly as a result of minimal trauma. The gold standard for a displaced PHF is plate osteosynthesis. However, many of these fractures fail due to a lack of medial calcar support. Our study evaluated the addition of a fibular strut graft on the effect on these fractures.

Methodology

A retrospective review was performed over 12 months in our institute. We included patients with three- or four-part PHFs with osteoporosis and medial calcar comminution. The patients were operated on using PHILOS plating with fibular autograft and were followed up for radiological and functional outcomes.

Results

A total of 13 patients, of whom 6 were males, were included in the study. The average age was 53.69 (41-63) years, and 9 patients had four 4-part fractures. All patients achieved union, with an average time to union being 5.2 months. There was a significant improvement in both the Quick Disabilities of the Arm, Shoulder, and Hand (QuickDASH) score and the visual analog scale (VAS) at one year. All patients achieved a near normal range of motion, and X-ray evaluation showed all fractures to be anatomically reduced at one year, with only one patient with varus alignment of 11 degrees.

Conclusions

Our study found the addition of fibular strut autograft in patients with proximal humeral fractures with medial calcar comminution as an adequate augmentation with good functional outcomes and minimal complications.

## Introduction

Proximal humerus fractures (PHFs) are relatively common, accounting for about 5% of all fractures [1,2]. In older individuals, especially those with conditions like osteoporosis or osteopenia, PHFs are the third most common type of fracture, with an estimated incidence of approximately 105 cases per 100,000 people each year [3-5]. These fractures commonly result from low-energy trauma in older adults, while they typically require more forceful impacts in younger individuals.

When treating PHFs, plate osteosynthesis is considered the gold standard [6], done using the proximal humerus internal locking osteosynthesis (PHILOS) plate. However, patients with osteopenic bones and those with loss of medial support suffer from loss of reduction, which is the most common cause for revision surgery [7]. Other than that screw penetration and varus collapse are common complications associated with this technique. Multiple studies have found that a lack of medial calcar support is one of the most important predictors of failure after surgical fixation of PHFs [8-14]. 

To address these challenges multiple techniques have been evaluated such as varus impaction of fracture fragments and use of calcar support screws. However, these techniques do not reconstruct the medial support and may result in inferior outcomes as a nonanatomical reduction is accepted.

We have evaluated the technique of using a fibular autograft to recreate the medial calcar support in osteopenic fractures with medial comminution using the PHILOS plating system and have studied its effects on clinical, radiological, and functional outcomes.

## Materials and methods

We conducted a retrospective study at the Central Institute of Orthopedics, Safdarjung Hospital, New Delhi, from April 2021 to April 2022. A total of 15 patients (6 males and 9 females) who had unilateral displaced proximal humeral fractures were included [15]. These patients were treated using the PHILOS plate with intramedullary fibular strut graft augmentation.

Our inclusion criteria consisted of adults with closed, displaced three- and four-part proximal humeral fractures, specifically those with a disrupted medial hinge and significant metaphyseal comminution or insufficient osseous contact, and a Dual-Energy X-ray Absorptiometry (DEXA) score of >2.5 Z-score from a DEXA study either performed preoperatively or within one year before trauma. We excluded patients with open fractures, pathological fractures, irreparable head and/or tuberosity fragments, and two-part fractures from the study.

Before surgery, we conducted a preoperative assessment using plain anteroposterior (AP) and lateral X-rays, along with computed tomography (CT) scans to assess displacement.

The patients were followed up for one year and were assessed clinically and radiologically at one month, six months, and one year.

Clinical evaluation included range of motion, pain assessment using the visual analog scale (VAS), and the Quick Disabilities of the Arm, Shoulder, and Hand (QuickDASH) score [16,17].

Radiographic assessments included the head shaft angle in the AP view, with Jiang et al. recommending that varus or valgus alignment of less than 5 degrees in the AP view be considered anatomical [18].

**Figure 1:**
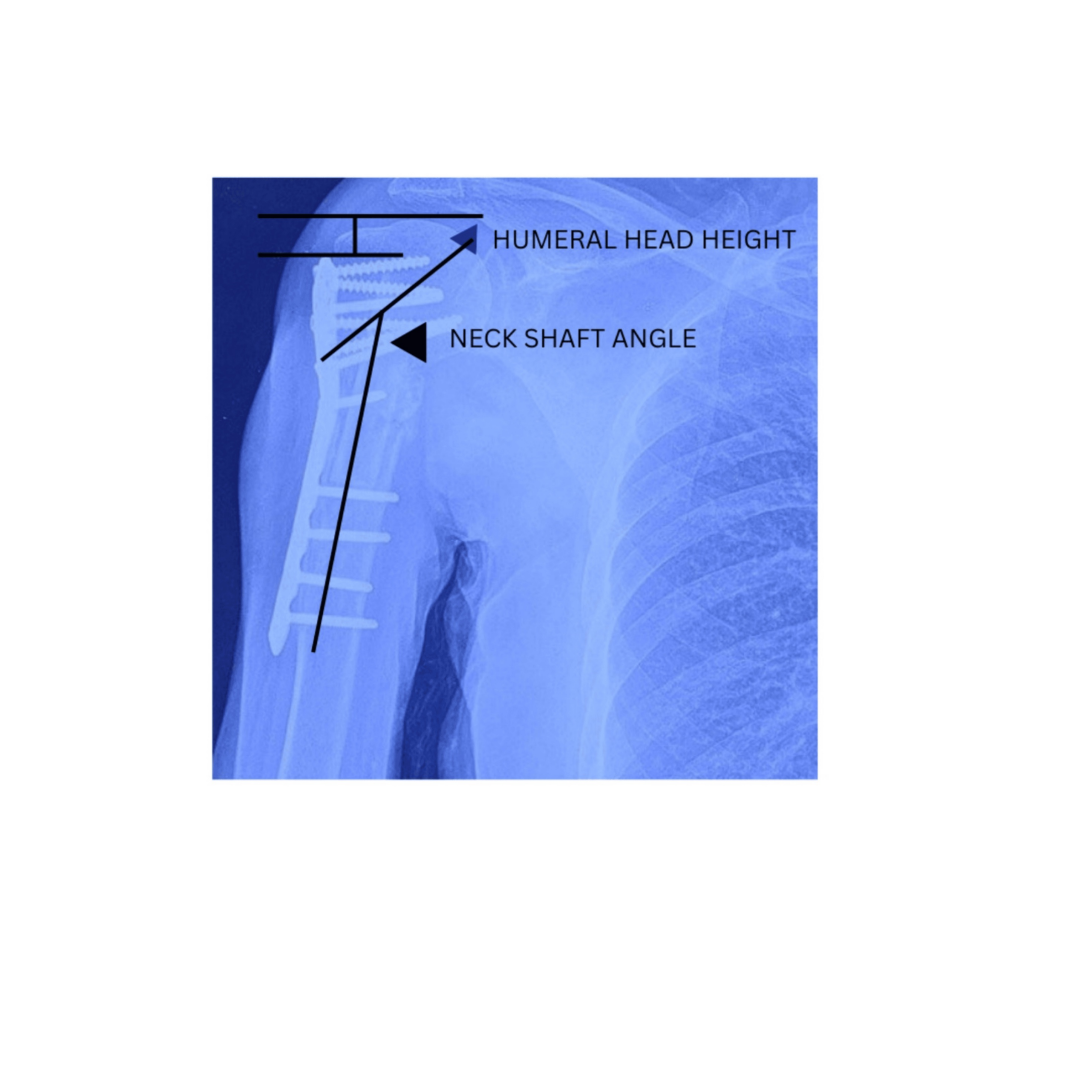
Measurement of the humeral head height and the neck-shaft angle.

The *humeral head height* relative to the plate was measured on each radiograph, both initially and at the final follow-up, to analyze potential loss of reduction. This measurement involved drawing two lines perpendicular to the plate shaft: one at the top edge of the plate and the other at the superior edge of the humeral head [19]. The distance between these lines, designated as the head height, was measured. A change in humeral head height exceeding 3 mm was classified as loss of reduction (Figure 1) [12].

Complications, such as fracture collapse, screw penetration, avascular necrosis, peri-implant fracture, infection, and the need for additional surgery, were documented during the study [20].

Surgical technique

The patient was positioned in *beach chair* posture on a radiolucent table and received general anesthesia. All patients were operated on via a standard deltopectoral approach. Identification and retraction of the cephalic vein were performed either medially or laterally based on ease of retraction and visualization. The conjoint tendon was retracted medially, and further exposure was achieved through limited reflection of the deltoid and pectoralis major insertion. Preservation of the biceps tendon was ensured.

Rotator cuff tendons were tagged using Ethibond No. 5 sutures to allow traction and gain control of the greater and lesser tuberosity fragments. Threaded K-wires were inserted into the humeral head fragment to aid in initial reduction, acting as a *joystick*.

In all cases, a fibular autograft was taken from an ipsilateral leg using two mini-incisions over the middle third of the lateral aspect of the leg directly over the fibula and was used to enhance the medial calcar. The fibular autograft was appropriately sized and shaped using an oscillating saw or osteotome and then inserted into the canal, maximizing medialization to the calcar region for indirect reduction of the medial margin. It was then pushed superiorly into the humeral head to help elevate it and allow indirect reduction using the strut graft.

Subsequently, the PHILOS plate was applied to the lateral humeral shaft distal to the fibular autograft, and its correct placement was confirmed using image intensification. Following anatomical reduction, locking screws were used to transfix the humeral head via the fibular strut graft to the shaft and the plate. The sutures previously secured in the rotator cuff were passed through the plate and tied off. The incision was closed in layers over a drain, and negative suction was applied. The drain was removed 48 hours later, and the arm was immobilized in a universal shoulder immobilizer. Prophylactic intravenous antibiotics were administered.

Postoperatively, pendulum exercises were initiated on the first day, and shoulder mobilization began with passive assisted exercises for the first four weeks, transitioning to active exercises for the next four weeks. After adequate active range of motion was achieved, strengthening was initiated. After 12 weeks, patients were allowed to resume activities of daily living was adequate strength and range of motion were achieved.

## Results

**Figure 2:**
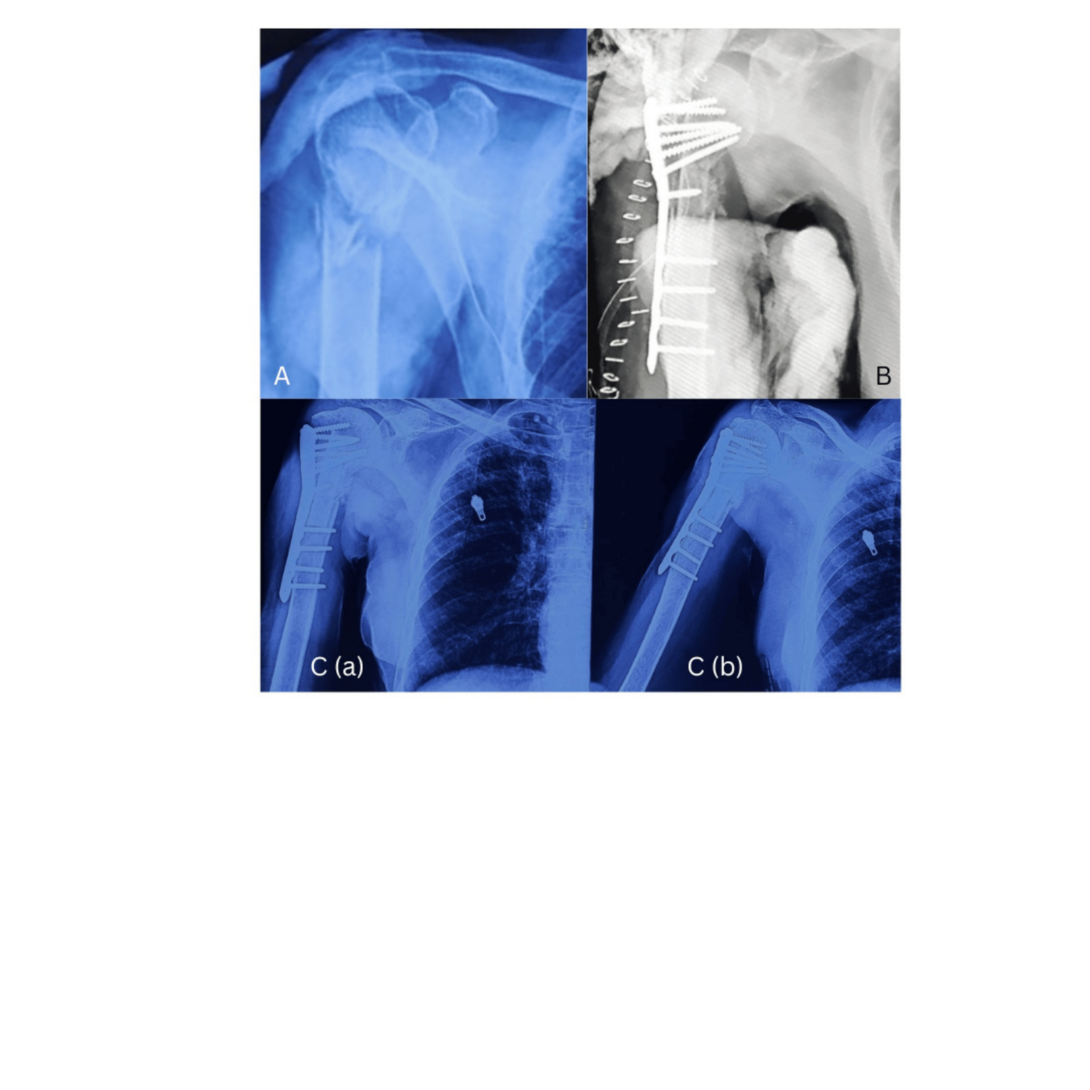
(A) Preoperative X-ray; (B) immediate postoperative X-ray; (C) (a and b) 12 months postoperative X-ray.

A total of 13 patients underwent surgery, of which six were male, with an average age of 53.69 years (range: 41-63 years). Nine patients had a four-part fracture according to the Neer classification, and the rest had a three-part fracture. All patients had neglected injuries, with an average duration between the date of injury and the time of surgery of 8.3 months (range: 6-12 months). All patients achieved union, with an average time for union being 5.2 months (4-6 months) (Figure 2; Table 1).

**Table 1:** Demographic distribution of patients. Data are presented as mean and standard deviation (SD).

	Mean	SD
Age (years)	53.69	7.36
Range of motion (degrees)		
Flexion	144.2	14.97
Extension	35.76	5.34
Abduction	146.92	14.36
Adduction	16.92	6.62
External rotation	69.6	8.02
Internal rotation	65.38	8.28
Time to union (months)	5.62	0.51

No patients were lost to follow-up. All fractures were found to have healed clinically and radiologically. 

The average QuickDASH score at six-month follow-up was 36.18 (22.72-43.18), which progressively decreased to an average of 9.05 (2.5-15.9) by the one-year mark. It was observed that there was a significant improvement in the QuickDASH score from six months to one year (*P* = 0.00). There was no effect of the delay of surgery on the QuickDASH score outcome, both at six and 12 months (Table 2).

**Table 2:** QuickDASH score at six months and one year. A *P*-value of <0.05 is considered significant. A paired *t*-test was used for comparison between the two. QuickDASH, Quick Disabilities of the Arm, Shoulder and Hand

QuickDASH	Mean	SD	*t*-value and *P*-value
6 months	36.18	7.03	*t* = 10.6176; *P* = 0.00001
12 months	9.05	4.79	

In terms of pain assessment using the VAS, the average score was 1.46 (0-3) points at the one-year follow-up. VAS score significantly improved from six months to one year (*P* = 0.0004), with no effect of the delay of surgery on the outcome (Table 3).

**Table 3:** VAS score at six and 12 months. A paired t-test was used with significance kept at *P* < 0.05. VAS, visual analog scale

VAS score	Mean	SD	*t*-value and *P*-value
6 months	3	0.7	*t* = 4.3818; *P* = 0.0009
12 months	1.46	0.96	

**Figure 3:**
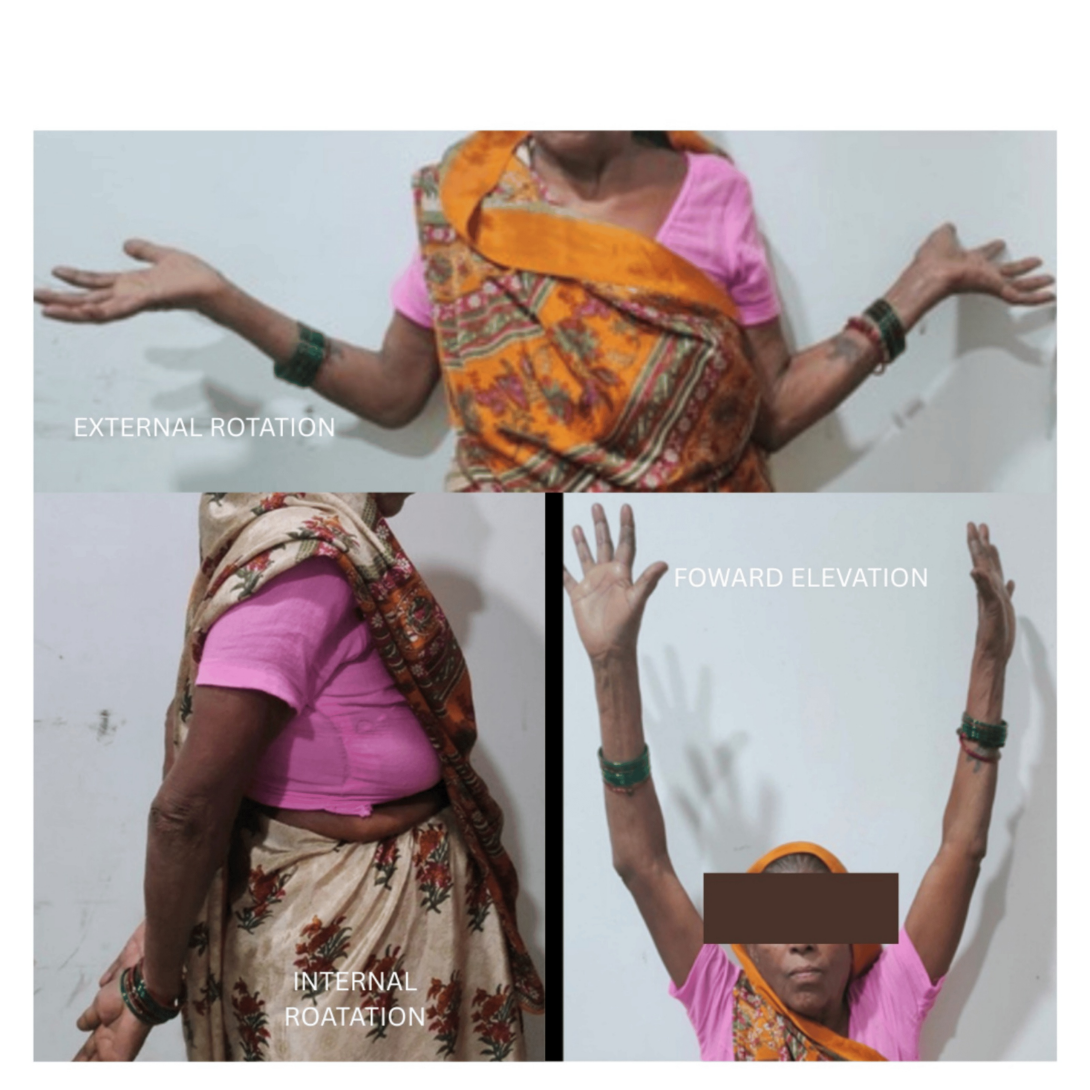
The rotations achieved at 12 months postoperatively. The right side is the operated side.

Regarding shoulder range of motion, the mean measurements were flexion: 144.23 degrees (ranging from 110 to 165 degrees), extension: 35.76 degrees (ranging from 30 to 45 degrees), internal rotation: 65.38 degrees (ranging from 55 to 75 degrees), external rotation: 69.61 degrees (ranging from 60 to 85 degrees), abduction: 146.92 degrees (ranging from 120 to 160 degrees) (Figure 3).

Alignment in the AP view was considered anatomical in 13 patients, while one patient showed a slight varus alignment of 11 degrees. There were no instances of humeral head collapse or screw penetration into the articular surface among any of the patients. The change in humeral head height from postoperative day 1 to the final one-year follow-up X-ray averaged 0.4 mm (with a maximum of 2.0 mm).

All cases showed restored medial cortical continuity, and the fibular autograft demonstrated progressive integration into the proximal humerus. One patient experienced a superficial infection shortly after surgery, which was successfully treated with oral antibiotics for 2 weeks without any complications.

None of the patients exhibited major complications such as subacromial impingement, neurovascular injury, implant loosening, or osteonecrosis of the humeral head.

No patient suffered from any complications from the fibular harvest site except one who had a postoperative hematoma, which was drained in the early postoperative period.

These results indicate an improvement in functional outcomes, reduced pain levels, and favorable shoulder range of motion and radiological outcome at the one-year follow-up assessment.

## Discussion

We assessed the effectiveness of a novel approach to treating unstable proximal humeral fractures using a combination of a locking plate and fibular autograft. Our findings indicate positive clinical outcomes for all patients, with no instances of collapse of humeral head or screw penetrance into the joint surface. Additionally, the fractures healed without any alteration in humeral height, as observed in X-rays taken immediately after surgery and during the final follow-up.

Gardner et al. [14] were the first researchers to publish findings on this technique in medical literature, demonstrating positive outcomes with all seven fractures healing without reduction loss or fixation instability. Subsequent research by Neviaser et al. showed minimal reduction loss (2.6%), no instances of screw cut-out (0%), and low rates of osteonecrosis (2.6%) in a study involving 38 patients with displaced proximal humeral fractures. They utilized locking plate fixation combined with an endosteal strut augment [21]. Biomechanical assessments indicated that incorporating medial support through an intramedullary fibular graft and angular stable fixation enhanced overall bone-implant construct rigidity and reduced migration of the humeral head fragment compared to using the locking plate alone [19,20,22-24].

Chow et al. [23] conducted experiments using cadaveric specimens to demonstrate the effectiveness of fibular allograft augmentation in strengthening a locking plate against repetitive varus loading. They observed that none of the augmented constructs failed before reaching 25,000 cycles, whereas 6 out of 8 non-augmented constructs failed at an average of 6,604 cycles [19,23]. Additionally, their tests with increasing loads showed significantly higher maximum failure loads and stiffness in the locking plate combined with intramedullary fibular grafts compared to using the locking plate alone [22].

Chui et al. [25] retrospectively evaluated the use of fibular allograft to augment fixation of proximal humeral fractures with a locking compression plate (LCP). They compared two groups of patients, with one group using only the LCP plate and the other group having both the Fibular allograft and the LCP. The Humeral head height loss was significantly more in the patients with only LCP fixation compared to those augmented with fibular allograft, and similar observations were found in the neck shaft angle, indicating higher loss of fixation in patients with only LCP. Functional outcome scores were also better in the Fibular allograft group, though not reaching significance. The complication rates were also higher in those where only LCP was used for fixation, indicating that allograft use protects the construct and prevents loss of fixation. 

Panchal et al. [26] also retrospectively evaluated 18 patients from the 50- to 70-year age group managed using a proximal humerus locking plate with fibular autograft. Postoperatively, all patients achieved union, with an average time to union of eight months, and demonstrated good to fair outcomes based on the American Shoulder and Elbow Surgeons (ASES) score and the University of California, Los Angeles (UCLA) shoulder rating scale. The neck-shaft angle on average was 120 to 130 degrees, with no patients having humeral head height loss >4 mm.

Osterhoff et al. [27] recently investigated a similar approach using synthetic bone. They augmented a proximal humerus locking plate with an intramedullary fibular allograft strut and observed significantly reduced intercyclic fragment motion, overall fragment motion, and residual gap-distance deformation after 400 cycles of loading in the group with the fibular graft compared to the standard technique. Moreover, the augmented construct exhibited greater stiffness and a higher ultimate load to failure. 

Our results support the utilization of fibular autograft and locking plates in managing proximal humerus fractures with medial comminution. This approach helps restore the integrity of the medial column, provides support to the humeral head, and aids in maintaining reduction until the fracture heals [19]. Additionally, the fibular autograft serves as both an indirect tool for fracture reduction and a mechanical support for the humeral head.

However, the conclusions are limited by a small sample size and lack of a control group, and further studies with a larger patient population are necessary.

## Conclusions

The combination of a locking plate with fibular graft augmentation proves to be a dependable and promising method for supporting the humeral head and sustaining reduction in the treatment of proximal humeral fractures with medial comminution. We recommend employing this technique specifically for such fractures to restore the medial column's integrity, provide necessary support to the humeral head, and ensure maintenance of reduction until the fracture heals. This approach has the potential to reduce common complications associated with proximal humeral locking plates and enables early initiation of aggressive physiotherapy.
